# Incidence of bovine neonatal pancytopenia in 243 farms in Germany

**DOI:** 10.1186/s12917-016-0857-7

**Published:** 2016-10-07

**Authors:** Frederike Reichmann, Annette Pfitzner, Guenter Rademacher, Elke Schwedinger, Klaus Cussler, Carola M. Sauter-Louis

**Affiliations:** 1 Clinic for Ruminants with Ambulatory and Herd Health Services at the Centre for Clinical Veterinary Medicine, LMU Munich, Sonnenstrasse 16, 85764 Oberschleissheim, Germany; 2 Paul-Ehrlich-Institut, Paul-Ehrlich-Straße 51-59, 63225 Langen, Germany; 3 Institute of Epidemiology, Friedrich-Loeffler-Institute, Suedufer 10, 17493 Greifswald, Isle of Riems Germany

**Keywords:** BNP, Calf, Epidemiology, Haemorrhagic diathesis

## Abstract

**Background:**

Several research groups from different European countries have worked on the aetiopathogenesis of bovine neonatal pancytopenia (BNP) and an association between the use of the vaccine PregSure BVD (Pfizer, Germany) and the development of this haemorrhagic disease was confirmed. Because BNP is not a notifiable disease, it is difficult to obtain information on its incidence. Based on pharmacovigilance (PhV) data, which are the only officially available data at the national level, the incidence of BNP is considered low. However, voluntary reporting of the disease can lead to underreporting.

To gain more insight into the incidence of BNP among the affected herds, an epidemiological study was performed, which focused on 243 farms in Germany with cases of BNP. Farmers were asked to report the occurrence of BNP, including the number of cases, which allowed calculation of incidence in the affected herds. Matching such data with the registered cases in the National PhV System (NPhVS) gave us an opportunity to assess the extent of BNP underreporting.

**Results:**

On 243 farms, a total of 1195 calves younger than 4 weeks with haemorrhagic diathesis were registered. In 58 % of the reports, a diagnosis of BNP was confirmed by blood analysis and or by necropsy. The number of cases observed on individual farms ranged from 1 to 80. Based on these results, the incidence of BNP on affected farms ranged from 0.3 to 15.2 % (median 2.9 %). The maximal incidence in the year with the highest number of BNP calves ranged between 0.4 and 18.6 % (median 3.3 %). Comparing the number of cases registered in the NPhVS to the numbers found in this study revealed considerable underreporting to the national database: only 44 % of the farms and 41 % of the BNP calves included in the study were registered in the NPhVS.

**Conclusions:**

In spite of the opportunity to report BNP calves to the Paul-Ehrlich-Institut (Langen, Germany), the estimated number of undetected BNP cases is remarkably high. However, even if the revealed substantial underreporting is taken into account, the incidence of BNP is low. Nevertheless, the incidence on some affected farms is very high, resulting in considerable financial losses that should not be underestimated. Although the exact pathomechanism of BNP at the molecular level is still not known, its incidence is clearly declining following withdrawal of PregSure BVD from the market.

**Electronic supplementary material:**

The online version of this article (doi:10.1186/s12917-016-0857-7) contains supplementary material, which is available to authorized users.

## Background

First cases of bovine neonatal pancytopenia (BNP) were reported in Germany in 2006 and then in several other European countries and New Zealand [1]. The work of several research groups in various European countries has yielded important information about the aetiology and pathogenesis:

The consumption of colostrum-containing alloreactive antibodies plays a crucial role in the development of BNP [2, 3]. Vaccination against bovine viral diarrhoea virus (BVDV) was suspected to be correlated with the pathogenesis of BNP [4]. Intensive investigations identified the inactivated vaccine PregSure BVD (Pfizer, Germany) as a major risk factor for developing BNP [5–7]. In some cows, the PregSure BVD vaccine induces alloreactive antibodies that target antigens derived from bovine cells used for vaccine production [8]. Owing to the consumption of colostrum from these cows, some calves and foster calves develop thrombocytopenia, leukocytopenia, and panmyelophthisis [8–10]. As a consequence, affected calves show bleeding symptoms and most often, immunosuppression [2, 11, 12]. Owing to the absence of a promising therapy for BNP, only about 10 % of affected calves survive [4, 10, 11]. A subclinical course of the disease has been described [13]. The most important differential diagnoses for the clinical signs of BNP are the haemorrhagic form of BVD (type 2), a severe septicaemia, and other haemorrhagic diatheses. The haemorrhagic form of BVD is very uncommon in Germany; in 95 % of BVDV infections, the virus is type 1 [14]. Furthermore, bleeding disorders of cattle are relatively rare. If these disorders occur, they generally happen not only in calves within the first 4 weeks of age, but at all ages. No other haemorrhagic diatheses show similar processes to BNP [15]. Classic septicaemia affects newborn calves between 2 and 6 days of age, and is attributed to other diseases. Bleeding signs in septicaemic calves are possibly due to capillary fragility or thrombocytopenia, but they are less typical signs [16].

There is a remarkable difference in the incidence of BNP among different regions within a country [17]. In addition to this geographical difference, the number of cases per herd also varies [2, 4, 12]. BNP is not a notifiable disease and, therefore, exact figures about its occurrence are not available. Because the occurrence of BNP is believed to be related to the use of a vaccine, each case should be reported as a possible adverse event within the pharmacovigilance (PhV) scheme. Veterinary surgeons and farmers have the opportunity to report BNP cases to their national competent authority, which for Germany, is the Paul-Ehrlich-Institut in Langen. The agency recorded 4623 cases in Europe (date of download: 28.02.2011), including 3040 cases from Germany..

However, there is currently no information available as to what degree these numbers truly reflect the BNP incidence. Furthermore, little information exists about the incidence within an individual herd; most often, the information is based on investigations on single farms. According to reported results, within-herd incidence are no higher than 10 % [8].

The aim of our study was to provide an overview of the occurrence of BNP based on 243 affected farms in Germany. The incidence of BNP was calculated and the data of this study were compared with cases registered in the National PhV System (NPhVS) to identify to what extent BNP cases were reported within the PhV scheme.

## Methods

### Participating farms

Since the first case of BNP, affected calves have been registered in a database at the Clinic for Ruminants with Ambulatory and Herd Health Services, LMU Munich (Oberschleissheim, Germany). Calls to encourage registration from all over Germany were published in *Deutsches Tieraerzteblatt*, a journal of the German Veterinary Association. The database included 310 farms on January 3, 2011. Farms were eligible as case farms if they had one or more confirmed cases of BNP by necropsy (panmyelophthisis) and or by blood analysis (thrombocytopenia [less than 200 × 10^9^ platelets (PLT)/L] and leukocytopenia [less than 4 × 10^9^ white blood cells (WBC)/L]). Blood analyses were performed by the laboratory of the Clinic for Ruminants with Ambulatory and Herd Health Services, LMU Munich. Necropsies were performed by one of the following institutions: Bavarian Authority for Health and Food Safety in Oberschleißheim or Erlangen, Bavarian Animal Health Service in Poing, Institute of Veterinary Pathology, Center for Clinical veterinary Medicine, LMU Munich or Animal Health service in Fellbach, Baden-Wuerttemberg.

Twenty-seven farms were excluded, because the blood analysis of affected calves with haemorrhagic diathesis revealed only thrombocytopenia or leukocytopenia. Further, farms which only reported on BNP calves without any confirmed case, were excluded (*n* = 28). In total, 255 farms with at least one confirmed case of BNP were selected from the database.

### Interview

Farmers were interviewed by telephone between January 3 and March 31, 2011. The interviews were conducted in a uniform fashion by one veterinarian (FR) at the Clinic for Ruminants. The veterinarian was trained prior to the interviews and instructed to avoid leading questions. The following data were collected for the years from 2005 to 2011:

- Number of cows per year
- Number of calves born per year
- Breed
- Type of farm (dairy farm or beef farm)
- Number of observed calves younger than 4 weeks of age with haemorrhagic diathesis each year
- Proportion of confirmed cases of calves with BNP among the observed calves with haemorrhagic diathesis
- Use of PregSure BVD.

One year was defined as the 12 month period from the first of January until the 31st of December. During the interview, the answers were recorded on paper. In case that the herd manager needed more time to look up the information, the farm was contacted a second time. If the farmer did not have any information about the vaccine that was used to vaccinate against BVDV, the veterinarian was contacted. If the farm had changed the veterinarian during the considered timeframe, the former veterinarian was called to get the information needed. Only farms that used PregSure BVD were included in the analysis. The questionnaire is available as Additional file 1.

### Incidence of BNP at the herd level

The total incidence on each farm was calculated as:

$$ \mathrm{Total}\ \mathrm{incidence}\ \left(\%\right) = \frac{\mathrm{Number}\ \mathrm{of}\ \mathrm{B}\mathrm{N}\mathrm{P}\ \mathrm{calves}}{\begin{array}{l}\mathrm{Average}\ \mathrm{number}\ \mathrm{of}\ \mathrm{calves}\ \mathrm{born}\ \mathrm{per}\ \mathrm{year} \times \\ {}\mathrm{Years}\ \mathrm{with}\ \mathrm{observed}\ \mathrm{B}\mathrm{N}\mathrm{P}\ \mathrm{cases}\end{array}}\times 100 $$

The considered time period for each farm was not equal due to the fact that BNP occurred on some farms over several years and on other farms only within 1 year. The aim of the study was to evaluate the incidence of BNP on farms while the disease was prevalent on the farm. Using the calculation above, allowed the comparison of incidences across farms. In addition, the maximal incidence was calculated, which was related to the year with the highest number of calves with BNP on the farm:

$$ \mathrm{Maximal}\ \mathrm{in}\mathrm{cidence}\ \left(\%\right) = \frac{\begin{array}{l}\mathrm{Highest}\ \mathrm{number}\ \mathrm{of}\ \mathrm{calves}\kern0.252em \mathrm{with}\ \mathrm{B}\mathrm{N}\mathrm{P}\\ {}\mathrm{in}\ \mathrm{one}\ \mathrm{specific}\ \mathrm{year}\end{array}}{\mathrm{Number}\ \mathrm{calves}\ \mathrm{born}\ \mathrm{in}\ \mathrm{that}\ \mathrm{year}}\times 100 $$

### Data matching between the NPhVS and the clinic for ruminants

Data matching was used to compare the number of farms and cases of BNP registered in the national PhV database at the Paul-Ehrlich-Institut against the numbers calculated in our study. Therefore, the first digits of the postal zip codes and the initials of the farmers were compared. Farms and cases not listed in the NPhVS were counted.

### Descriptive and statistical analyses

The geographical distribution of the farms affected by BNP was illustrated in a map created in ArcGIS software (ESRI, version 9.3). A descriptive statistical analysis was conducted using Excel version 13.0 (Microsoft Inc., USA) and SPSS for Windows version 19 (SPSS Inc., USA). Binomial confidence intervals were calculated in Excel using the method of Agresti and Coull [18].

## Results

### Participating farms

**Fig. 1:**
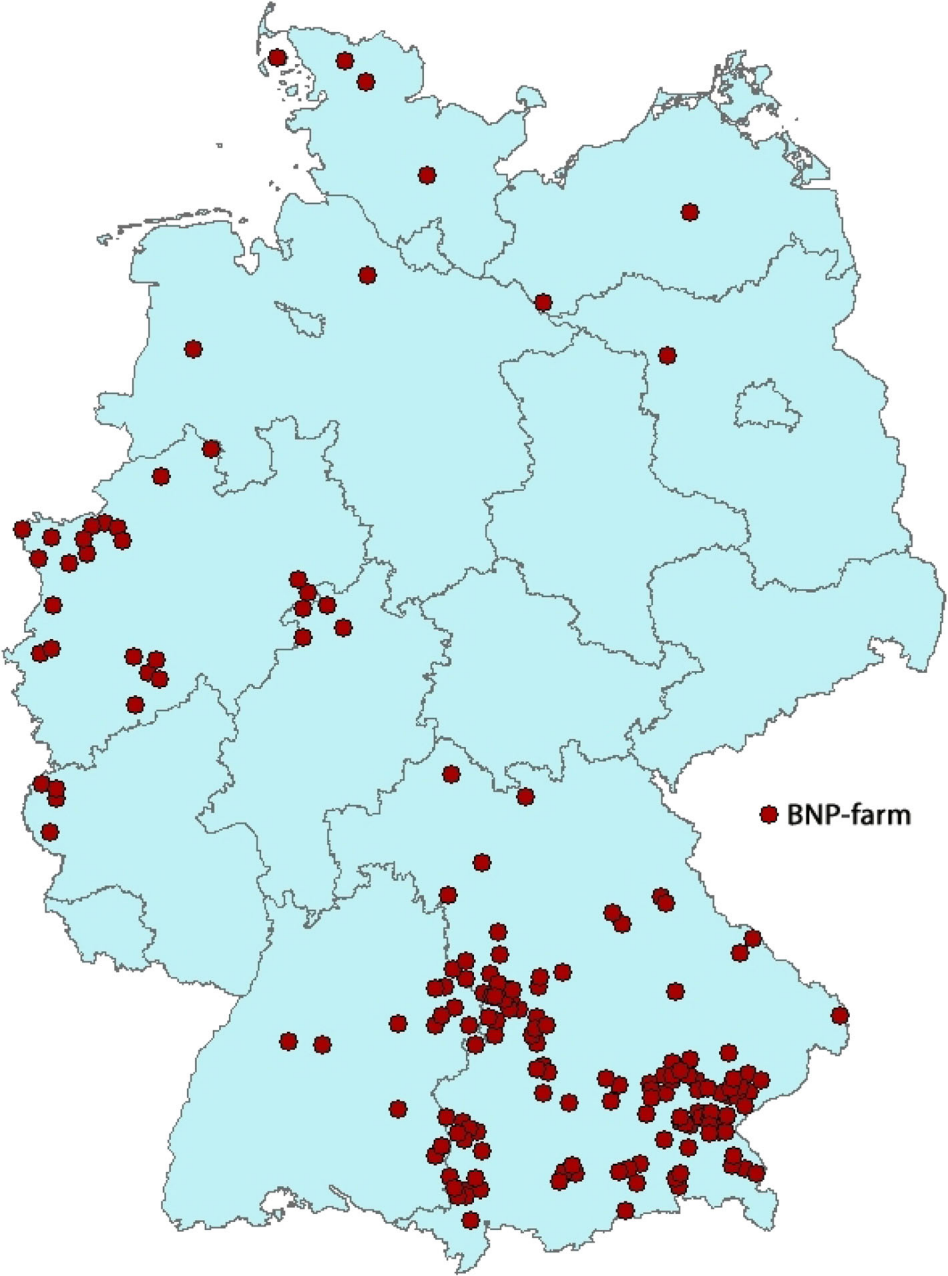
Geographical distribution of participating farms with bovine neonatal pancytopenia (BNP) (*n* = 243) The farms were listed in a database of the Clinic for Ruminants with Ambulatory and Herd Health Services at the Centre for Clinical Veterinary Medicine, LMU Munich (Oberschleissheim, Germany). At least one calf younger than four weeks of age was registered on each farm, with haemorrhagic diathesis that was confirmed as BNP by blood analysis and or necropsy

Two of the 255 contacted farmers were not willing to give an interview and one farmer had stopped farming, thus they were excluded from the analysis. Nine farms were excluded, because the herd was either not vaccinated against BVDV or PregSure BVD was not used. Finally, 243 farms could be included and farmers were available and willing to participate in the study. The veterinarians were always available to complete the data about the occurrence of BNP calves and the vaccination against BVDV, in case they were needed. Most of the farms (*n* = 241) were dairy farms with 168 having German Fleckvieh (70 %), 47 farms with Holstein-Frisian (19 %), 12 farms with Brown Swiss (5 %), nine farms with Red Holstein (4 %) and five farms with German Fleckvieh crossbred (2 %). Two farmers had a cow-calf operation with Limousin and German Fleckvieh, respectively. The geographical distribution of the participating farms is shown in Fig. 1. Of the 243 farms, 76 % were located in Bavaria and 24 % were in eight other federal states of Germany. The herd size ranged from 14 to 950 cows (average 67).

### Total number of calves with haemorrhagic diathesis (four weeks old or younger)

**Fig. 2:**
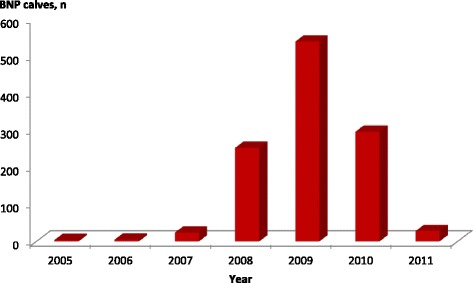
Number of observed cases of bovine neonatal pancytopenia (BNP) on 241 farms between 2005 and 2011. The study was finished on March 31, 2011. BNP cases that occurred afterwards in 2011 were not recorded. BNP cases of two farms are not shown, because the exact distribution of BNP calves over the years were not available

During the interviews, the 243 farmers reported 1195 newborn calves with a bleeding disorder occurring between January 1, 2005, and March 31, 2011 (Fig. 2). The first four cases were observed in 2005 on three different farms. However, a remarkable increase in the number of calves being observed with BNP was not noted until 2008, when 253 cases were observed on 88 farms. The year 2009 had the highest number of observed cases: 68 farms that had previously reported BNP cases and 120 new farms were affected and recorded. A total of 539 calves had clinical signs of haemorrhagic diathesis during their first month of life. After 2009, the number of cases declined on all participating farms.

### Number of cases observed per farm

**Fig. 3:**
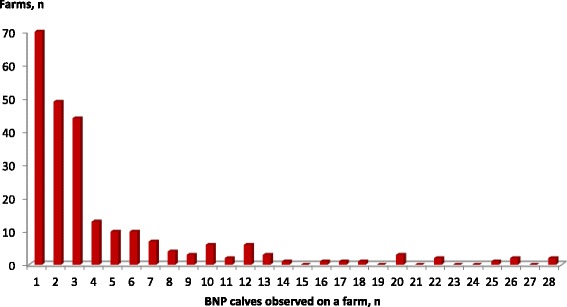
Number of farms that have observed a particular number of bovine neonatal pancytopenia (BNP) cases between 2005 and March 2011. One farm with 50 cases and another one with 80 are not included in the figure, because they are outliers

The number of newborn calves with haemorrhagic diathesis on each farm is illustrated in Fig. 3, excluding two farms with 50 and 80 cases, because they are outliers.

BNP cases were registered during only 1 year on nearly one-half of the farms (44 %). Thirty-five percent of the 241 farmers noted calves with BNP during two of the years, 36 farmers during three of the year, and another 12 farmers during four of the years. One interviewed owner reported cases during 5 years and another one during 6 years. Some farms had a year without having calves with BNP between the years with cases of BNP.

In 58 % of the registered cases, BNP was confirmed either by blood analysis and or by necropsy. In 42 % of the cases, farmers did not initiate an examination to clarify the aetiology of the bleeding disorder.

### Overall incidence and incidence of BNP at the herd level

The incidence could not be calculated for two farms, because the farmers did not remember the exact distribution of BNP calves over the years. Regarding the whole timeframe (January 1, 2005 to March 31, 2011), the overall incidence of BNP on 243 farms added up to 1 %. In the year with the highest number of cases (2009), the incidence across all farms was 2.8 %.

**Fig. 4:**
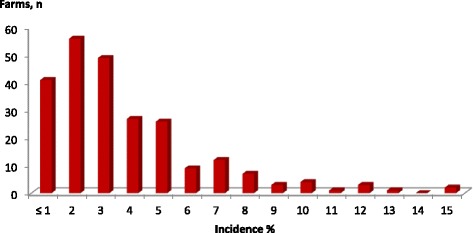
Within-herd incidence of bovine neonatal pancytopenia (BNP). The incidence was calculated using the number of BNP calves on 241 affected farms from January 1, 2005, to March 31, 2011. The rate ranged from 0.3 to 15.2 % (median 2.9 %). The incidence could not be calculated for two farms, because the exact distribution of BNP cases over the years was not available

The incidence of BNP within the 241 farms ranged between 0.3 and 15.2 % (median 2.9 %). The incidence distribution is shown in Fig. 4. The maximal incidence in the year with the highest number of BNP calves ranged from 0.4 to 18.6 % (median 3.3 %). The difference between the total incidence and the maximal incidence was particularly marked on farms with BNP cases scattered over several years, including 1 year with an irregularly high number of calves with BNP. On some of those farms, the maximal incidence was two to three times higher than the total incidence throughout the surveyed period.

### Data matching between the NPhVS and the clinic for ruminants

Data matching between the two databases revealed 701 of 1195 cases (59 %) were not registered in the NPhVS, but were noted in the interviews conducted in our study. A total of 137 of the 243 participating farms (56 %) had not reported any cases to the NPhVS. Only 41 % of the interviewed Bavarian farmers and only 33 % of the 759 BNP calves from Bavaria in our study were registered with the NPhVS. Ten of 12 farms from Baden-Wuerttemberg reported their cases to the NPhVS, but only 24 of 47 interviewed farmers from the other federal states of Germany reported their cases. The data matching results are shown in Table 1.

**Table 1:** Results of data matching between the Clinic for Ruminants with Ambulatory and Herd Health Services at the Centre for Clinical Veterinary Medicine, LMU Munich (Oberschleissheim, Germany), and the National Pharmacovigilance System (NPhVS): Comparison of bovine neonatal pancytopenia (BNP) farms and the number of BNP cases on those farms

Federal State	Farms reported to the Clinic for Ruminants	Farms from the study registered at the NPhVS	Proportion of NPhVS-registered farms in % (Confidence Interval)	Total BNP cases on the farms of the LMU study	BNP cases on the farms of the LMU study registered at the NPhVS	Proportion of NPhVS-registered BNP cases in % (Confidence Interval)
Bavaria	184	75	40.76 (33.92–47.98)	759	248	32.67 (29.43–36.09)
Baden-Wuerttemberg	12	10	83.33 (55.20–95.30)	67	54	80.6 (69.58–88.29)
Brandenburg	1	1	100.00 (20.65–100.00)	6	6	100 (60.97–100.00)
Hesse	5	4	80.00 (37.55–96.38)	47	7	14.89 (7.14–27.69)
Lower Saxony	1	0	0.00 (0.00–79.35)	2	0	0.00 (0.00–65.76)
North Rhine-Westphalia	26	14	53.85 (35.46–71.24)	242	133	54.96 (48.66–61.1)
Mecklenburg-Vorpommern	2	2	100.00 (34.24–100.00)	39	32	82.05 (67.33–91.02)
Schleswig-Holstein	6	3	50.00 (18.76–81.24)	19	9	47.37 (27.33–68.29)
Rhineland-Palatinate	6	0	0.00 (0.00–39.03)	14	0	0.00 (0.00–21.53)

## Discussion

### Incidence of BNP at the herd level

One of the main objectives of this study was to identify the incidence of BNP at the herd level. For the calculation of the incidence the average number of calves born on the farm was available. This could have introduced a bias, as stillborn calves would not have been included in the denominator of the calculation. Using the average number of calves born on the farm also could have introduced a bias, which we cannot exclude. However, all farmers included in the study had reported that their herd size had been stable over the considered time period, therefore we are confident that the number of calves born over several years did not vary considerably. BNP affects calves during their first 4 weeks of life. Male Holstein-Frisian and Red Holstein calves are usually sold at an age of two weeks, thus it is possible that some cases of BNP were missed when BNP occurred after the calves were sold to another farm. However, most of the participating farms (77 %) had German Fleckvieh, crossbred or Brown Swiss. Male calves from those farms are typically not sold before they have reached a certain age or weight and do not leave the farm during the time when BNP may occur.

The highest incidence reported in a herd from our study (19 %) was substantially higher than the incidence rates reported in other studies, which was up to only 10 % [8]. Although the BNP incidence was low on most of the affected farms, some herds were obviously more severely affected. The incidence of BNP varies markedly among different countries and also among the federal states of Germany [4, 6]. The farms enrolled in our study were from nine federal states of Germany. However, most of the farms with cases of BNP were localized in Bavaria. Kasonta et al. [17] investigated the influence of different patterns of PregSure BVD vaccinations on the incidence of BNP. Their data showed that different vaccination regimens resulted in differences in alloreactive antibody concentrations. Therefore, the differences in incidence rates among federal states can potentially be explained by the different vaccination protocols that exist. Apart from the varying incidence among the federal states of Germany, we found considerable differences among the participating farms within the federal states. Presumably, the use of different vaccination regimens at the herd level is the reason for that observation, but other factors (e.g. colostrum management) could have had an influence on the incidence as well.

The present study shows a remarkable increase of BNP cases in 2008, reaching a peak in 2009. Although PregSure BVD was licensed in 2004, an increased BNP incidence was not noted until 2007. The same phenomenon was observed in New Zealand: the first cases of BNP occurred there in 2011, which was 3 years after the introduction of PregSure BVD [19]. Kasonta et al. [17] interpreted the temporal delay as being associated with the repeated immunizations that are required for creating a titre of alloreactive antibodies high enough to induce clinical BNP in the calves.

Various reasons exist for the decline of BNP cases on farms in our study since 2010. Owing to increased knowledge concerning aetiopathogenesis, farmers adapted their management practices, especially colostrum and vaccination management [20]. The safest way to prevent further BNP cases is to dispose of colostrum coming from known ‘BNP dams’ and to feed calves colostrum from cows that have not been vaccinated with PregSure BVD. Before PregSure BVD was withdrawn from the German market in April 2010, many farmers had already switched to a different BVD vaccine or discontinued vaccination against the BVDV. Nevertheless, calves with BNP are still observed on some farms, but in much smaller numbers [21]. Therefore, the antibody titre seems to remain high for a long time, even though PregSure BVD is no longer used. It is uncertain how much longer further cases of BNP will occur, but they will most likely stop when the last PregSure BVD-vaccinated cows are removed.

### Data matching between the two databases

Another objective of our study was to investigate whether the number of calves with BNP registered at the NPhVS reflects the real number of cases in Germany. More than one-half of the cases and farms had not been listed in the NPhVS database of the Paul-Ehrlich-Institut. Three-hundred cases of BNP were reported in our study that occurred before 2009, which was the year when BNP was recognized as a potential serious adverse effect of PregSure BVD. A retrospective report of these cases was technically possible, but seems unlikely to happen, so most of these cases are most likely not in the database. Although veterinarians are requested to report adverse events in accordance with the veterinary medical association’s professional code of conduct, they and the farmers do not directly benefit from reporting cases to the NPhVS. Thus, they are increasingly reluctant to fill in reporting questionnaires. Although we discovered a remarkable extent of underreporting, considering the population of 4.3 million dairy cows in Germany [22], the overall incidence is still very low.

### Economic relevance

The severity of economic losses in affected herds due to medical treatments of BNP calves, costs for the veterinarian, and especially case fatalities, is difficult to estimate. However, an appreciable pecuniary loss can be assumed for farms with high incidence. In addition, less obvious losses could be caused by subclinical BNP cases in which calves could be more susceptible to other diseases, especially infections due to immunosuppression. Thus, in an epidemiological study, 2.5 % of control calves had thrombocytopenia and leucocytopenia without clinical signs of BNP [15], and another research group induced subclinical BNP in 20 % of calves after feeding with colostrum from ‘BNP dams’ [23].

### Confirmation of BNP via blood analysis or necropsy

To reduce the bias of misclassification and, thus, maximize the probability of including farms with actual cases, only farms with at least one confirmed case of BNP were included in this study. Therefore, we excluded some farms from the study because even though the farmers had observed calves with signs consistent with BNP within their herds, they did not initiate an examination to verify the aetiology.

As reflected by the high proportion (58 %) of cases that were confirmed by haematology and or necropsy, veterinary practitioners demonstrated a great commitment to elucidate the previously unknown aetiology of this disease.

The spectacular clinical signs that are most often seen in affected calves, and the severe process of BNP are very similar among cases. Therefore, it can be assumed that affected farmers and their veterinarians are aware of the disease pattern and that most of the calves included in this study without disease confirmation likely did have BNP. Some cases of BNP were probably missed at the very beginning of the occurrence when farmers and veterinarians were not aware of the disease or in case of sudden death. Furthermore it is not possible to get any information about subclinical cases of BNP within a herd without blood sampling the calves, thus subclinical BNP could not be taken into account.

Because there are no advantages (e.g. compensation or specific treatments and better prognoses for the calves after disease diagnosis) for farmers and veterinarians in confirming additional cases, the proportion of investigated cases will decline and reports on the occurrence of BNP to official agencies will become less common over the years.

## Conclusions

The number of calves with BNP in Germany is higher than officially assumed. Many cases have not been registered as PhV reports at the NPhVS, and we revealed a remarkable underreporting of the disease.

The incidence of BNP varies considerably, not only among federal states, but also among affected herds within one area. Considerable financial losses can be assumed, especially on farms with a high incidence.

Considering the whole bovine population of Germany, the incidence of BNP is low, even when accounting for the fact that many affected calves have not been registered.

Due to the well-advanced clarification of the aetiopathogenesis of BNP and the suspension of the license for PregSure BVD, the peak incidence for this disease has passed. However, a few cases of BNP still occur. It is likely that cases of BNP will occur until the last dam vaccinated with PregSure BVD has left the farms.

## Additional file

Additional file 1: Questionnaire. Farmers were interviewed by telephone using this questionnaire. (DOCX 16 kb)

## Abbreviations

BNP: Bovine Neonatal Pancytopenia
BVD: Bovine Viral Diarrhoea
BVDV: Bovine Viral Diarrhoea Virus
NPhVS: National Pharmacovigilance System
PLT: Platelets
WBC: White Blood Cells
