# Supplementary material for: Incidence of bovine neonatal pancytopenia in 243 farms in Germany
Source: BMC Vet Res. 2016 Oct 7;12:220. doi: 10.1186/s12917-016-0857-7 (PMC5054546; doi:10.1186/s12917-016-0857-7)
Supplement: Additional file 1: — Questionnaire. Farmers were interviewed by telephone using this questionnaire. (DOCX 16 kb) [file 12917_2016_857_MOESM1_ESM.docx]

Questionnaire

Farm

Name: ______________________________________

Address: ______________________________________

______________________________________

Date of Interview ____________________

Type of farm: Dairy farm

Beef farm

| **Year** | **2011** | **2010** | **2009** | **2008** | **2007** | **2006** | **2005** |
| --- | --- | --- | --- | --- | --- | --- | --- |
| **Average number of cows** |  |  |  |  |  |  |  |
| **Number of calves born** |  |  |  |  |  |  |  |
| **Number of calves (≤ 4 weeks) with haemorrhagic diathesis** |  |  |  |  |  |  |  |
| **Confirmation of BNP via blood analysis (number of calves)** |  |  |  |  |  |  |  |
| **Confirmation of BNP via necropsy (number of calves)** |  |  |  |  |  |  |  |
| **Use of PregSure BVD**  **Y=yes N=no** |  |  |  |  |  |  |  |
